# Molecular cloning and characterization of a nuclear androgen receptor activated by 11-ketotestosterone

**DOI:** 10.1186/1477-7827-3-37

**Published:** 2005-08-17

**Authors:** Per-Erik Olsson, A Håkan Berg, Jonas von Hofsten, Birgitta Grahn, Anna Hellqvist, Anders Larsson, Johnny Karlsson, Carina Modig, Bertil Borg, Peter Thomas

**Affiliations:** 1 Department of Natural Science, Unit of Molecular Biology, Örebro University, SE-701 82 Örebro, Sweden; 2 Department of Marine Science, University of Texas Marine Science Institute, University of Texas, Port Aransas, Texas 78373, USA; 3 Department of Molecular Biology, Umeå University, SE-901 87 Umeå, Sweden; 4 Department of Zoology, Stockholm University, SE-106 91 Stockholm, Sweden

## Abstract

Although 11-ketotestosterone is a potent androgen and induces male secondary sex characteristics in many teleosts, androgen receptors with high binding affinity for 11-ketotestosterone or preferential activation by 11-ketotestosterone have not been identified. So, the mechanism by which 11-ketotestosterone exhibits such high potency remains unclear. Recently we cloned the cDNA of an 11-ketotestosterone regulated protein, spiggin, from three-spined stickleback renal tissue. As spiggin is the only identified gene product regulated by 11-ketotestosterone, the stickleback kidney is ideal for determination of the mechanism of 11-ketotestosterone gene regulation. A single androgen receptor gene with two splicing variants, belonging to the androgen receptor-β subfamily was cloned from stickleback kidney. A high affinity, saturable, single class of androgen specific binding sites, with the characteristics of an androgen receptor, was identified in renal cytosolic and nuclear fractions. Measurement of ligand binding moieties in the cytosolic and nuclear fractions as well as to the recombinant receptor revealed lower affinity for 11-ketotestosterone than for dihydrotestosterone. Treatment with different androgens did not up-regulate androgen receptor mRNA level or increase receptor abundance, suggesting that auto-regulation is not involved in differential ligand activation. However, comparison of the trans-activation potential of the stickleback androgen receptor with the human androgen receptor, in both human HepG2 cells and zebrafish ZFL cells, revealed preferential activation by 11-ketotestosterone of the stickleback receptor, but not of the human receptor. These findings demonstrate the presence of a receptor preferentially activated by 11-ketotestosterone in the three-spined stickleback, so far the only one known in any animal.

## Introduction

Androgens have critical physiological roles in male sexual differentiation and in the development of male secondary sex characteristics. Androgens primarily mediate their actions through interactions with receptors belonging to the steroid hormone receptor super-family. Androgen receptors (AR) have been identified in many vertebrates and demonstrate similar binding characteristics in the various species investigated. Most vertebrates have one AR with high specificity for 5α-dihydrotestosterone (DHT). However, some teleosts have two AR with high binding affinities for either testosterone (T) or DHT [1,2]. 11-ketotestosterone (KT) is a major androgen in many teleosts and it often occurs at higher levels in the circulation and has a higher potency in inducing male reproductive functions than other androgens [3]. Although several AR have been isolated and characterized from teleost species, none of these have characteristics expected in a specific KT receptor. Consequently, an explanation for the high androgenic potency of KT in teleosts is currently not available.

AR have been cloned and characterized from several teleosts, including Japanese eel (*Anguilla japonica*), rainbow trout (*Oncorhynchus mykiss*), tilapia (*Oreochromis niloticus*) and red seabream (*Pagrus major*) [4-7]. Rainbow trout, tilapia and Japanese eel have two AR isoforms and while only one isoform (ARα) is a functional AR in rainbow trout, both Japanese eel AR are functional receptors [6,7]. Following transfection of human embryonic kidney 293 cells with either of the Japanese eel AR cDNAs it was observed that KT and DHT were equally potent activators of both AR isoforms, while T was significantly less potent in activating an MMTV-LTR driven luciferase reporter vector [4,6]. In contrast, the red sea bream AR was equally activated by T and KT via an MMTV promoter system in transfected COS-7 cells [5]. Similarly, a rainbow trout ARα reporter system in a carp EPC cell line was equally activated by DHT, KT and T [8]. Thus, none of the previously cloned AR genes show any preference for KT in trans-activation assays. A lack of specificity for KT in androgen signaling pathways was also suggested by androgen binding studies on tissues from Atlantic croaker (*Micropogonias undulatus*), kelp bass (*Paralabrax clathratus*), rainbow trout and goldfish (*Carassius auratus*) [1,2,8-10]. Based on tissue specific binding profiles there are two forms of AR in Atlantic croaker and kelp bass. One isoform, AR1, shows the highest binding affinity for T followed by DHT and KT while the other, AR2, shows the highest affinity for DHT followed by T and KT [1]. A limitation of the previous studies was that no KT-regulated gene had been identified in any of the studied species, and therefore no definite conclusion regarding endogenous gene regulation by KT could be drawn.

Hypertrophy of the kidneys and the production of a glue, spiggin, used in nest building by the kidneys of male three-spined sticklebacks (*Gasterosteus aculeatus*) during the breeding season, is currently the clearest example of a male reproductive process induced by KT [3,11]. Although treatment of stickleback with a variety of androgens can induce the kidney hypertrophy normally observed during the breeding season, KT is by far the most potent inducer of kidney hypertrophy and spiggin production [12,13]. These findings are of physiological importance because KT is the major plasma androgen during the breeding season in male stickleback [14]. However, a previous study showing that [^3^H]-KT was equally displaced from stickleback kidney tissue fragments by unlabelled KT or DHT provided no evidence for the presence of a specific KT AR in this species [15]. Thus, even in sticklebacks, where a specific KT-regulated gene product has been identified, the mechanism by which KT exhibits high androgenic potency remains unclear.

Due to the well-known regulation of spiggin by KT in the three-spined stickleback, the stickleback kidney is an ideal system in which to determine the mechanism by which KT regulates gene expression in teleosts and to determine the possible involvement of specific KT receptors in this signaling pathway. The binding characteristics of stickleback kidney cytosolic and nuclear preparations were comprehensively investigated in the present study to determine the androgen specificity of the AR(s) in this species. Furthermore, AR was cloned from stickleback kidney RNA and the molecular structures of two splicing variants, ARβ1 and ARβ2, were characterized. While the investigations of the binding affinities of androgens for the stickleback AR indicated that DHT bound better than KT to the receptor, comparison of the trans-activation potency of the stickleback AR with the human AR showed that KT was more potent at down-stream gene activation than DHT, in transfected HepG2 and ZFL cells, in the presence of the stickleback AR but not of the human AR.

## Experimental

### Materials

[^3^H]-DHT (92.84 Ci/mmol) and [^3^H]-KT (98.05 Ci/mmol) were purchased from New England Nuclear (Boston, MA, USA) and stored at -20°C. The unlabeled steroids were purchased from either Steraloids, Inc. (Wilton, NH, USA) or from Sigma Chemical Company (St. Louis, MO, USA). All radiolabelled steroids were stored in 95% ethanol at -20°C. Chemicals and salts used for making the buffers were purchased from Sigma and from Fisher Scientific (Pittsburgh, PA, USA). The scintillation cocktail was a mixture of 4 L toluene, 16 g PPO (7,5-diphenyl-oxazole), and 0.4 g POPOP (1,4-bis [5-phenyl-2-oxazolyl]-benzene).

#### Fish Maintenance

Adult stickleback were caught in Öresund and routinely housed in large aquaria containing brackish water (0.5% salinity) at 20°C under a photoperiod of 16:8 h light:dark (LD). A total of 300 male and female sticklebacks were used in the present study. The fish ranged in weight between 1 and 3 g. The water was aerated and filtered and the bottom was covered with sand. The fish were fed red midge larvae daily. Kidneys from males that had been brought into breeding condition by exposure to 20°C under a photoperiod of 16:8 h LD were used for the characterization of the AR. For the experimental treatments, the fish were initially in non-breeding condition. For the spiggin-experiment post-breeding females were used, these had been exposed to LD 16:8 and 17°C for a few months. For the other experimental treatments, fish were caught in non-reproductive seasons and housed under low temperature (9°C) and a photoperiod of LD 8:16 until a few days before treatment when they were put into high temperature and long photoperiod in order to acclimate. These investigations were approved by the Stockholm Northern Animal Experiment Ethical Committee (permit N 185/00).

### Tissue sampling and steroid treatments

Steroid treatments were performed using females, as their kidneys do not undergo hypertrophy under natural conditions, and castrated males. The fish were anesthetized with 0.1% (v/v) 2-phenoxyethanol (Sigma, St. Louis, MO, USA) and implanted intraperitoneally with Silclear silicone tubing (10-mm length, 0.6-mm inner diameter, 1.2-mm outer diameter) containing steroids dissolved in cocoa butter. The steroids used were 11-ketoandrostenedione (KA), DHT, T and 17β-estradiol (E2) (1 or 25 μg μl^-1^). KA was used as this steroid is converted into KT by the fish [14]. Tubes containing cocoa butter alone were used as controls. Following implantation the fish were maintained in 50-liter aquaria containing brackish water (0.5% salinity) at 20°C under LD 16:8 h. At sampling fish were decapitated and the kidneys were excised, frozen using liquid nitrogen, and stored at -70°C. A number of experiments were performed as follows. Spiggin mRNA was measured in post-breeding females that were dissected 2 days following implantation of tubes. Cytosolic androgen-receptors were measured in females and in castrated males. Males were castrated under anesthesia as above, by making a 1.5 mm long incision into the abdominal cavity on each side and removing the testes using forceps. The females and the castrated males were implanted with control and high dose tubes and dissected after 6 days. AR mRNA was studied in females implanted for 12 h, 2 and 16 days (separate experiments) with control and high dose tubes and in males castrated as above and implanted for 10 days with control tubes and high doses of KA or E2.

### Reverse transcriptase-Polymerase Chain Reaction

Total RNA was extracted from a pooled sample of five mature male kidneys using Tri Reagent™ (Sigma). The cDNA was synthesized from 1 μg of total RNA using the First Strand cDNA Synthesis Kit (Amersham Pharmacia Biotech, Buckinghamshire, UK). Amplification reactions were assembled using oligonucleotides based upon conserved regions in teleost AR (GeneBank accession numbers: AB012095, AB012096, AB017158, AB023960, AF121257, AB025361 and AF326200). These oligonucleotides were: forward (5'-GGGAAACAGAAATACCTGTGTG-3') and reverse (5'-CTCTGCAATCATCTCTGGAAAG-3'). Amplification was conducted for 40 cycles at 94°C for 30 s, at 40°C for 1 min and at 72°C for 1 min using a PTC-200 Thermal Cycler (MJ Research, Waltham, MA, USA). Amplified products were ligated into pGEM^®^-T (Promega, Madison, WI, USA) and recombinant plasmids were isolated using the Wizard^® ^*Plus *SV Miniprep System (Promega). Cycle sequencing was performed using the DYEnamic ET Terminator Cycle Sequencing Premix Kit (Amersham Pharmacia Biotech, Piscataway, NJ, USA). The reactions were resolved on an ABI Prism™ 377 DNA Sequencer (Perkin-Elmer, Milano, Italy) and the data obtained were analyzed using EditView (Version 1.01) (Perkin-Elmer).

### cDNA library screening

A unidirectional cDNA library, against mature male kidney cDNA, constructed in Lambda ZAP Express^® ^(Stratagene, La Jolla, CA, USA) was used to screen for AR cDNA using a DIG-labeled AR cRNA probe. The screening was conducted according to the Lambda ZAP Express^® ^manual (Stratagene). DIG labeled anti-sense RNA probes were generated using the DIG RNA Labeling Kit (Roche, Mannheim, Germany). Hybridization was performed at 45°C overnight (O/N) in hybridization buffer (5 × SSC, 50% formamid, 0.02% SDS (w/v), 0,1% N-laurylsarcosine (w/v) and 2% blocking solution (w/v)) (Roche). Membranes were washed for 2 × 5 min in 2 × SSC and 0.1% (w/v) SDS at room temperature, and for 2 × 15 min in 0.2 × SSC, 0.1% (w/v) SDS at 68°C. Signals were detected using CSPD (Roche) and exposure of Hyperfilm™-MP film (Amersham Pharmacia Biotech, Buckinghamshire, England) and hybridization signals were visualized using a CURIX 60 Film Developer (AGFA-Gevaert AB, Kista, Sweden). Positive plaques were purified through four successive hybridization rounds, and individual clones were isolated by phagemid excision. Following sequence identification of the clones as AR, they were sequenced to completion by Cybergene AB (Huddinge, Sweden).

### Slot Blot Analysis

Total RNA was extracted using Tri Reagent™ (Sigma). Aliquots of 10 μg of total RNA were mixed with denaturing solution (6 × SSC, 7% (v/v) formaldehyde) and transferred onto a nylon membrane (Amersham Pharmacia Biotech) using a Minifold II Slot Blot Apparatus (Schleicher and Schuell, Keene, NH, USA). Membranes were probed using either a randomly primed [α-^32^P]-dCTP radiolabelled AR cDNA fragment (918 base pairs) that was isolated by reverse transcriptase-polymerase chain reaction and sequenced as above, or a randomly primed [α-^32^P]-dCTP radiolabelled spiggin-α cDNA. Hybridizations were performed at 65°C overnight (6 × SCC, 0.1% (w/v) SDS, 100 μg ml^-1 ^tRNA, and 5 × Denhardt's solution). The membranes were washed for 2 × 30 minutes at 42°C and 65°C in 0.1 × SCC, 0.1% (w/v) SDS whereafter Hyperfilm™-MP film was exposed at -70°C. The films were visualized using Curix 60 Film Developer. Following AR and spiggin mRNA determination the membranes were stripped and analyzed for 18S rRNA using a cDNA fragment as internal control. Each AR or spiggin transcript was then semi-quantified using Quantity ONE 4.2.3 (Bio-Rad, Laboratories, Inc, Hercules, CA, USA) in relation to its internal 18S transcript.

### Southern analyses of stickleback genomic DNA

Southern analyses were performed using 20 μg aliquots of male or female stickleback genomic DNA digested with 10 units of either *SacI*, *BamHI *or *EcoRI *at 37°C for 8 h according to Sambrook *et al*. [16]. Membranes were probed using [α^32^P]-dCTP radiolabelled partial cDNA encoding stickleback AR, which was isolated and sequenced as described above. Hybridizations were performed at 60°C O/N in 5 × SSC, 0.02% SDS (w/v), 0,1% N-laurylsarcosine (w/v) and 1% blocking solution (w/v) (Roche). Membranes were washed for 2 × 30 min periods at 42°C and 60°C in 0.1 × SSC, 0.1% (w/v) SDS. Membranes were exposed to Hyperfilm™-MP and were visualized as described above.

### Sequence similarity analysis

The amino acid sequence alignments were made using sequences obtained from the GenBank sequence data bank with the following accession numbers: Human, *Homo sapiens *AR (M34233); Mouse, *Mus musculus *AR (X53779); Rat, *Rattus norvegicus *AR (M20133); Zebra finch, *Taeniopygia guttata *AR (AF532914); African clawed toad, *Xenopus leavis *AR (U67129); Tilapia ARα (AB045211); Tilapia ARβ (AB045212); Burton's mouthbreeder AR, *Haplochromis burtoni *AR (AF121257); Burton's mouthbreeder ARβ (AY082342); Japanese eel ARα (AB023960); Japanese eel ARβ (AB025361); Red seabream AR (AB017158); Three-spined stickleback ARβ (AY247207); Goldfish AR (AY090897); rainbow trout ARα (AB012095); rainbow trout ARβ (AB012096); Japanese medaka, *Oryzias latipes *ARα (AB076399). The sequence alignments were performed using Omiga 2.0 (Oxford Molecular Ltd). A phylogenetic tree was constructed using Tree view (Version 1.6.2) [17] following the alignment using the Clustal W algorithm (Version 1.7) [18] based on the GenBank sequences used for the sequence alignment.

### Expression vector cloning

The excised AR pBK CMV clones were digested with *XbaI *and *EcoRI *and the inserts were gel purified prior to ligation into the pCMV-TNT vector (Promega) between *XbaI *and *EcoRI*. The obtained AR pCMV-TNT constructs were confirmed by sequencing. AR was expressed by introducing the pCMV-TNT into the TNT coupled reticulocyte lysate system (Promega) according to the manufacturer's instructions. The size of the expressed products was determined by addition of S^35^-methionin to the reticulocyte lysate system. The obtained protein suspension was analyzed on a denaturing 8% SDS PAGE and the sizes were determined using prestained SDS-PAGE broad range protein molecular weight standard (Bio-Rad) and a control luciferase protein of 62 kDa included in the reticulocyte lysate system (Promega). For use in binding assays AR was expressed in the absence of radioactive S^35^-methionein and stored at 4°C until further used in recombinant binding assay experiments.

### Sample preparation for AR assays

The excised kidney was weighed and thereafter frozen in liquid nitrogen prior to storage at -80°C. Following thawing the samples were homogenized in 10 volumes ice cold homogenization buffer (50 mM Tris-HCl, 10 mM sodium molybdate, 1 mM EDTA, 12 mM monothioglycerol and 10% glycerol (v/v), pH 7.4) by using a Polytron Tissuemizer (Tekmar, Cincinnati, OH, USA) a setting 7 for 10 seconds followed by five passes in a glass homogenizer. The homogenate was centrifuged at 2,500 × g for 15 min. The supernatant was transferred to an ultracentrifuge tube and spun at 150,000 × g for 60 min. The supernatant was charcoal stripped by mixing 10 ml of the supernatant with 2.5 ml Dextran-coated charcoal buffer (DCC) (50 mM Tris-HCl, 1 mM EDTA, 10% glycerol (v/v), 1% Norit-A charcoal (w/v) and 0,1% Dextran T-70 (w/v), pH 7.5) and centrifuging for 10 min at 6,000 × g to pellet the charcoal and obtain the cytosolic fraction. The cytosolic extract was further diluted 5 × with homogenization buffer prior to binding assay analysis. The crude nuclear pellet obtained from the initial 2,500 × g spin was washed three times in ice-cold wash buffer (10 mM Tris-HCl, 2 mM MgCl_2_, 2 mM monothioglycerol, 250 mM sucrose and 10% glycerol (v/v), pH 7.5). The resulting pellet was resuspended in 5 ml extraction buffer (homogenization buffer supplemented with 0.7 M KCl), and incubated for 60 min at 4°C with vortexing at 15 min intervals. The preparation was centrifuged at 150,000 × g for 60 min and the supernatant provided the nuclear fraction. The preparations were either used immediately or kept frozen at -80°C until assayed.

### Binding Assay

[^3^H]-DHT or [^3^H]-KT was dried under nitrogen, re-dissolved in 50 μl ice-cold homogenization buffer and added to assay tubes with or without a 100-fold excess of unlabeled DHT or KT. Aliquots (250 μl) of the nuclear or cytosolic preparations were added to each tube, the tubes were vortexed and incubated over night at 4°C. To stop the binding reaction, 250 μl DCC was added to each tube. The tubes were vortexed and the suspensions were incubated for 5 min at 4°C, before spinning at 3,000 × g at 4°C for 5 min. The supernatants were decanted into 7 ml scintillation vials, 5 ml of standard scintillation cocktail was added and the radioactivity in each sample was measured in a liquid scintillation counter (Beckman LS 6000SC, Beckman Instruments, Fullerton, CA). The procedure used for the expressed recombinant ARβ2 was modified as follows. [^3^H]-DHT was dried down under nitrogen, re-dissolved in 100 μl ice-cold homogenization buffer and added to assay tubes with or without a 1,000-fold excess of unlabeled DHT. The expressed proteins were diluted 1:100 and aliquots (100 μl) were added to each reaction tube. The tubes were vortexed and incubated over night at 4°C. To stop the binding reaction, 200 μl DCC was added to each tube. The tubes were vortexed and the suspension were incubated for 5 min at 4°C, before spinning at 3,000 × g at 4°C for 5 min. 250 μl of the supernatant was transferred into 5 ml scintillation vials and 4 ml of standard scintillation cocktail was added before the radioactivity in each sample was measured in a liquid scintillation counter (Wallac 1409, Wallac Oy, Turku, Finland).

### Saturation and Scatchard analysis

[^3^H]-DHT (0.42 – 18.2 nM) or [^3^H]-KT (0.42 – 15.4 nM) was added to each reaction tube with or without a 100-fold excess of unlabeled steroid. For the expressed AR, [^3^H]-DHT (2.5 – 42 nM) was added to each reaction tube with or without a 1000-fold excess of unlabeled steroid. Nuclear or cytosolic suspensions were incubated with steroids over night at 4°C. The reactions were terminated and the radioactivity measured as described above. Non-linear curve fitting procedures (GraphPad Prism, version 3.03, GraphPad Software Inc) were used to calculate the dissociation constant (K_d_) and to estimate the concentration of binding sites (B_max_) in the different suspensions.

### Association and Dissociation kinetics

To investigate the time needed to reach binding equilibrium, cytosolic preparations were incubated in 4 nM [^3^H]-DHT with or without 400 nM unlabeled steroid. The reaction was terminated at different time-points ranging between 1 min and 24 h. The specific binding at each time-point was determined as described above. In order to investigate the receptor dissociation time, the preparation were incubated over night with 4 nM [^3^H]-DHT with or without 100-fold excess of unlabelled DHT for determining non-specific binding, before adding 100-fold excess of unlabelled DHT, and binding was subsequently measured between 5 min and 28 h.

### Steroid specificity

The steroid specificity of receptor binding was examined using a competitive binding assay. Diluted protein suspension (1:100) were incubated over night at 4°C in tubes containing 1 nM [^3^H]-DHT with or without unlabelled steroid at end concentrations ranging between 1 pM and 100 μM. Steroids used were: KT; DHT; T; E2; 17,20β-dihydroxy-4-pregnen-3-one (17,20β-P); 17,20β,21-trihydroxy-4-pregnen-3-one, (20β-S) and cortisol (F). Following incubation, the reaction was terminated by addition of DCC and centrifugation and the specific binding in each tube was determined as described above.

### Cell culture and transfection assays

Two liver epithelial cell lines were used to test receptor activity. HepG2 cells (ATCC, Manassas, VA) were maintained in DMEM supplemented with 10% FCS and non-essential amino acids. ZFL cells (ATCC) were maintained in 50% L-15 Leibovitz, 15% H-12 Ham and 35% DMEM supplemented with 10% FCS (Gibco, Paisley, Scotland, UK) and 50 ng ml-1 EGF (Sigma). Cells were cultured at 37°C (HepG2) or 28°C (ZFL) in 5% CO_2_. The transfection experiments were initiated by replacing the tissue culture medium with fresh phenol-free medium supplemented with charcoal stripped FCS and the cells were seeded on 24-well plates. Transfections were performed at 90–95% confluence using 1,5 μl Lipofectamine 2000 (Invitrogen, Carlsbad, CA, USA) and 0,6 μg DNA per well. Transfected DNA contained 60 ng pRL (Promega), 270 ng androgen response element (ARE) luciferase reporter vector, 270 ng stickleback ARβ2 expression vector or 270 ng human AR expression vector (pCMVhAR) while 270 ng empty TNT cloning vector was used to control background luciferase levels following transfections. Two different ARE containing promoter constructs were tested, one containing 3 separate ARE (HRE; *slp*-ARU) and a second one construct (*slp*-HRE2) containing 4 copies of the ARE with high affinity for R1881, a synthetic androgen, and low affinity for dexamethasone, a synthetic glucocorticoid [19].

After 16 h the transfection medium was replaced with fresh phenol-free medium, supplemented with charcoal stripped FCS, containing different steroid hormones. The cells were exposed to the hormones for 40 h and luciferase levels were detected using the Dual Luciferase Assay kit (Promega) in a TD 20/20 Luminometer (Turner Designs, Sunnyvale, CA). For each cell line, transfection assay was performed for each concentration with n = 4 and the luciferase value of each assay were normalized to its corresponding *Renilla *luciferase activity. Each experiment was repeated a minimum of 3 times. The fold-induction is presented as luciferase values normalized against the control. The control levels were arbitrarily set to 1.0 for each cell line.

### Statistical analyses

All data from semi-quantitative analyses of mRNA are shown as AR in relation to 18S expression and presented as mean ± SD. Significance were determined using ANOVA and Student *t *test.

## Results

### Spiggin induction

**Figure 1:**
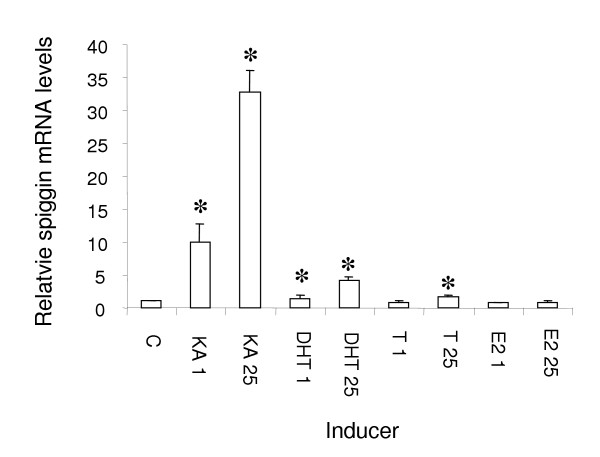
**Induction of spiggin mRNA in adult female stickleback kidney by different steroids**. KA, 11-ketoandrostenedione; DHT, 5α-dihydrotestosterone; T, testosterone; E2, 17β-estradiol (1 or 25 μg/μl in implants). All values represent the mean (±SD) values from 6 fishes. The mean value of the control group (C) was arbitrarily set to 1.00. Significant difference from the control is indicated by an asterisk (P < 0.01).

Implantation of females with 25 μg KA caused a 30-fold increase in spiggin mRNA levels. At this dose, KT was 10 times more effective than DHT and 50 times more effective than T at increasing kidney spiggin mRNA levels (Fig. 1). When implanted with 1 μg steroid a more pronounced difference was observed with KT being 23 times more potent than DHT and T not inducing spiggin production.

### AR cloning and characterization

**Figure 2:**
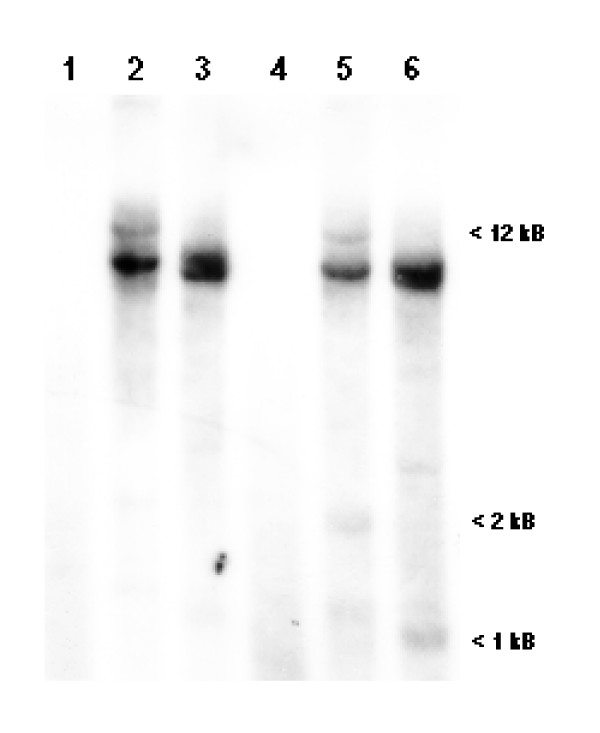
**Southern analysis of the AR locus**. Stickleback genomic DNA (20 μg), from one male (lanes 1–3) and one female (lanes 4–6) kidney, digested with *EcoRI *(lane 1 and 4), *SacI *(lane 2 and 5) and *BamHI *(lane 3 and 6). The position of molecular weight markers (kb) is given in the right margin.

A 918 base pair internal fragment of the three-spined stickleback AR was isolated by RT-PCR and used to screen a mature male three-spined stickleback kidney lambda ZAP Express cDNA library. Several positive clones were obtained and two different size transcripts were identified. One longer sequence (3168 bp) coded for a partial AR, ARβ2 (Gene Bank Accession no AY247206), and one shorter sequence (2515 bp) coded for a full length AR, ARβ1 (Gene Bank Accession no AY247207). The ARβ1 sequence contained a 322 bp 5'-UTR (untranslated region) including an in frame stop codon, a 2046 bp coding sequence and a 147 bp 3'-UTR, and the gene coded for a 682 amino acid AR (ARβ1) protein. The ARβ2 sequence differed from ARβ1 in that it contained additional amino acid sequence in the N-terminal region, coding for 735 amino acids and also contained a longer (961 bp) 3'-UTR. The 3'-UTR of both transcripts was identical up to the poly-A tail of the shorter UTR (147 bp), indicating the presence of two poly-adenylation sites in the stickleback AR gene. The sequence of both transcripts exhibited a conserved identity (100%) in overlapping regions at both the nucleotide and protein levels, thus indicating that both were derived from a single locus by alternative splicing. Southern analysis of genomic material from both male and female stickleback demonstrated a hybridization pattern that was compatible with the existence of a single ARβ gene (Fig. 2).

**Figure 3:**
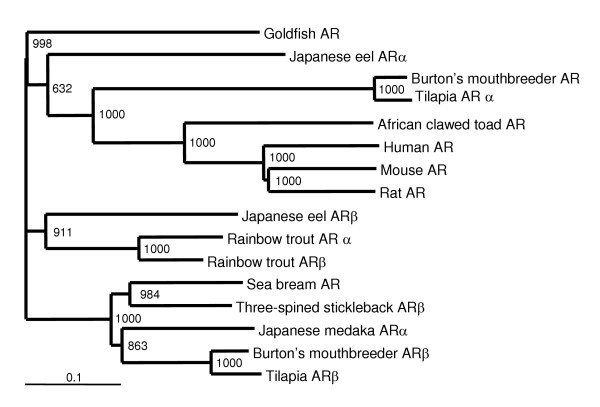
**Comparative sequence analysis of selected vertebrate AR proteins**. The tree was constructed using Tree View (Version 1.6.2) following alignment of the protein sequences by the Clustal W algorithm (Version 1.7). GenBank Accession Nos.: human AR (M34233); mouse AR (X53779); rat AR (M20133); African clawed toad AR (U67129); tilapia ARα (AB045211); tilapia ARβ (AB045212); Burton's mouthbreeder AR (AF121257); Burton's mouthbreeder ARβ (AY082342); Japanese eel ARα (AB023960); Japanese eel ARβ (AB025361); red seabream AR (AB017158); three-spined stickleback ARβ (AY247207); goldfish AR (AY090897); rainbow trout ARα (AB012095); rainbow trout ARβ (AB012096); Japanese medaka ARα (AB076399). The numbers at the base of each clade division represent bootstrap values after 1000 repeats. Scale bar represents 0.1 amino acid replacements per amino acid site.

**Figure 4:**
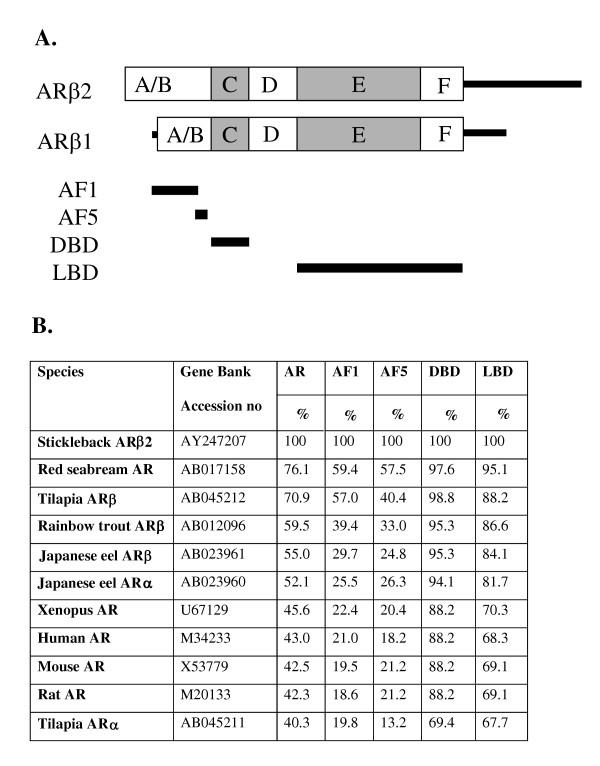
**Percentage similarity of AR and specific AR domains**. *A*, Schematic representation of the stickleback AR and the localization of specific domains. The AF1 (activator function 1) domain corresponds to amino acids (aa) 102–370 in the human AR. The AF5 (activator function 5) domain corresponds to aa 360–385 in the human AR. The DNA binding domain (DBD) corresponds to aa 550–635 in the human AR. The ligand binding domain (LBD) corresponds to aa 672–919 in the human AR. *B*, Percentage similarity to selected AR from different species.

Sequence comparison analysis defined the three-spined stickleback AR as an ARβ isotype clustered with other teleost ARβ isotypes (Fig. 3). The closest overall similarity was found with the red seabream AR (76.1%) and the tilapia ARβ (70.9%) (Fig. 4). Low similarity was observed with ARα isoforms and with the mammalian AR. Determination of sequence similarities between different domains showed a high conservation of the DNA-binding domain (DBD) and the ligand binding domain (LBD), while the N-terminal trans-activation domains (AF1 and AF5) were less conserved (Fig. 4). In both AF1 and AF5, the stickleback ARβ showed the highest similarity to red seabream AR and tilapia ARβ.

**Figure 5:**
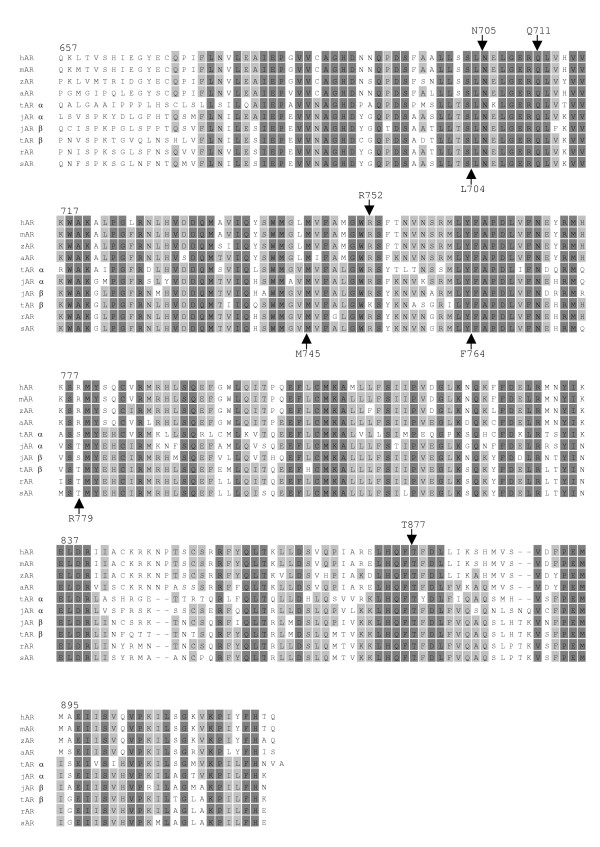
**Sequence alignment of the ligand-binding region of AR**. The alignment was performed using Omiga 2.0 (Oxford Molecular Ltd). Amino acid identity between sequences was illustrated with grey boxes and amino acid match with black boxes. Numbering is according to the human AR sequence. Amino acids (N705, Q711, R752, T877) with possible interaction with DHT in human AR are indicated by arrows from above. Amino acids with possible close contact to DHT (L704, M745 and F764) and of importance for ligand binding pocket architecture (R779) are indicated with arrows from below. GenBank Accession Nos.: Human AR (hAR) (M34233); Mouse AR (mAR) (X53779); Zebra finch AR (zAR) (AF532914); African clawed toad AR (aAR) (U67129); Tilapia ARα (tARα) (AB045211); Tilapia ARβ (t ARβ) (AB045212); Japanese eel ARα (jARα) (AB023960); Japanese eel ARβ (jARβ) (AB025361); Red seabream AR (rAR) (AB017158); Three-spined stickleback ARβ (sAR) (AY247207).

Alignment of the three-spined stickleback AR LBD with other AR showed that the sequences were highly conserved and that the four amino acids thought to be involved in direct ligand-interactions with DHT in human AR (N705, Q711, R752 and T877), or amino acids with possible close contact to DHT (L704, M745 and F764), were conserved in the stickleback [20]. An arginine, located at position 779 in human AR that has been suggested to be of importance for ligand binding pocket architecture [21], has been substituted with a threonine in the stickleback sequence (Fig 5).

**Figure 6:**
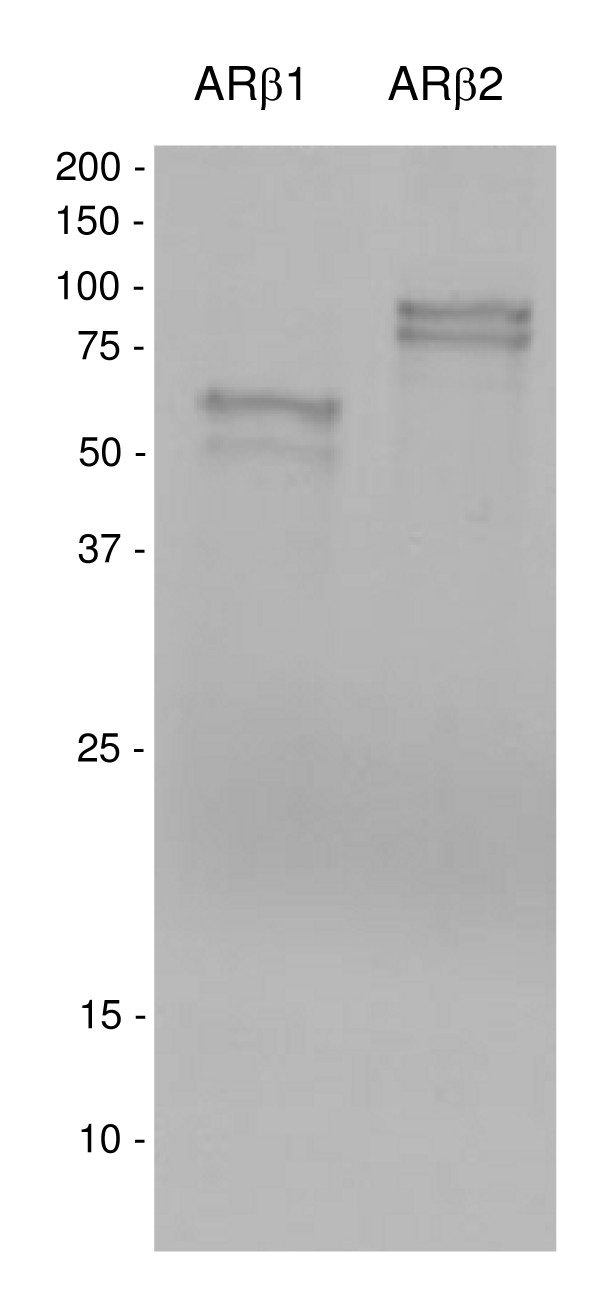
***In vitro *translation products of three-spined stickleback ARβ1 and β2**. AR was expressed by introducing the pCMV-TNT into the TNT Coupled Reticulocyte Lysate System. The size of the expressed products was determined by addition of S^35^-methionin to the reticulocyte lysate system. The protein suspensions were analyzed on a 8% SDS PAGE. Deduction of protein size from sequencing data yielded a expected size of 76.6 kDa for ARβ1 and 82.2 kDa for ARβ2. Estimation of translation product sizes resulted in 61 kDa and 52 kDa for ARβ1 and 87 kDa and 75 kDa for ARβ2.

Both AR splicing variants were re-cloned into the pCMV-TNT expression vector in order to produce AR *in vitro *for binding assays and for expression in HepG2 and ZFL cells. S^35^-Met labeled AR was separated on a SDS-polyacrylamide gel to determine the size of the expressed proteins. From the nucleotide sequence, ARβ1 was estimated to code for a 76.6 kD protein while ARβ2 was estimated to code for a 82.2 kD protein. Both AR migrated as double bands following electrophoresis and the transcript sizes were 52 and 61 kD for ARβ1 and 75 and 87 kD for ARβ2 (Fig. 6). These results indicate that ARβ2 was properly translated in the reticulocyte system, while ARβ1 appeared as a truncated translation product.

### AR binding characteristics

**Figure 7:**
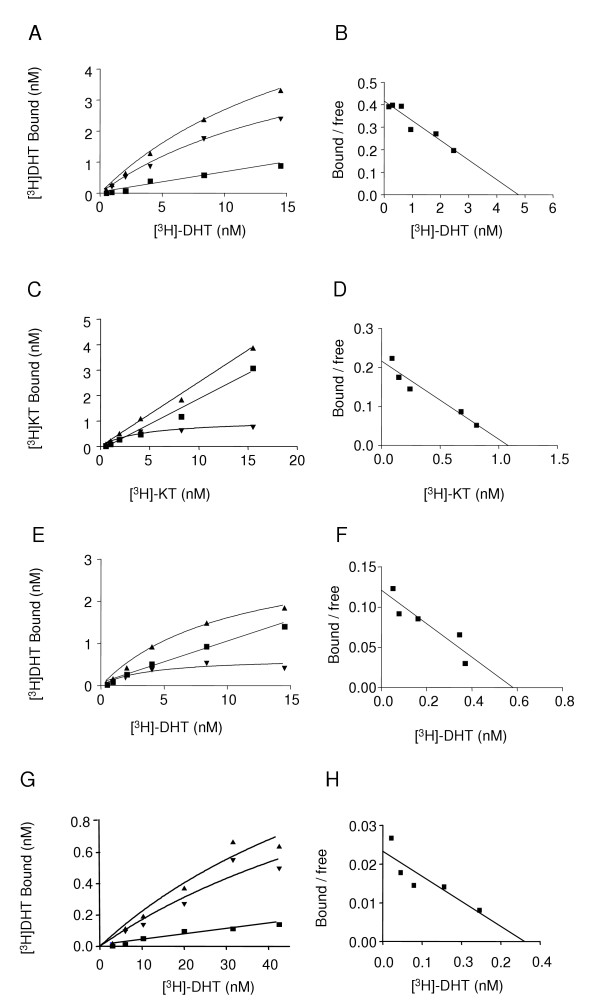
**Representative saturation curves and Scatchard analyses**. Saturation curves describing [^3^H]-DHT binding to AR in cytosolic fraction (A), [^3^H]-KT binding in cytosolic fraction (C), [^3^H]-DHT binding to AR in nuclear fraction (E) and [^3^H]-DHT binding to AR in reticulocyte lysate samples (G). Specific binding (▼) was determined by subtracting non-specific binding (■) from total binding (▲). Scatchard analyses of the specific binding of [^3^H]-DHT to cytosolic fraction (B), [^3^H]-KT to cytosolic fraction (D), of [^3^H]-DHT to nuclear fraction (F) and of [^3^H]-DHT to reticulocyte samples (H).

As a first step in the characterization of the AR binding characteristics in stickleback kidney, we determined the nature of interaction between ligand and receptor. Saturation ligand binding assays were performed on cytosolic, nuclear and membrane fractions of kidney extracts using either [^3^H]-DHT or -KT as tracer. In the initial studies we used [^3^H]-DHT to determine the presence of ARs in stickleback kidneys. High affinity and saturable binding was observed in both the cytosolic and nuclear fraction (Fig. 7A and 7E) while no specific binding to the membrane fraction was observed (data not shown). Next, using [^3^H]-KT we also observed high affinity and saturable binding sites in the kidney cytosol (Fig. 7C). Finally we used AR produced in the reticulocyte system to determine the characteristics of the cloned AR (Fig. 7G).

The saturation assay analysis, using [^3^H]-DHT as a tracer, was consistent with a single class of high affinity cytosolic AR with a K_d _of 18.7 ± 3.21 nM and a B_max _of 5.61 ± 0.63 pmol/g tissue (Fig. 7B). Specific binding of [^3^H]-DHT was also present in the nuclear fraction where a K_d _of 3,82 ± 0,26 nM and a B_max _of 0.67 ± 0.17 pmol/g tissue was observed (Fig. 7F). Saturation assay analysis, using [^3^H]-KT as a tracer, was also consistent with a single class of high affinity cytosolic AR with a K_d _of 4.44 ± 1.61 nM and a B_max _of 1.06 ± 0.15 pmol/g tissue (Fig. 7D). Analysis of the recombinant ARβ2 using [^3^H]-DHT as a tracer also showed high affinity specific binding to reticulocyte extract with a K_d _of 15.33 ± 2.50 nM and a B_max _of 0.324 ± 0.04 pmol/mg protein (Fig. 7H). The truncated translation product of ARβ1 did not give any measurable binding using the reticulocyte system and was therefore not further used in the present study.

**Figure 8:**
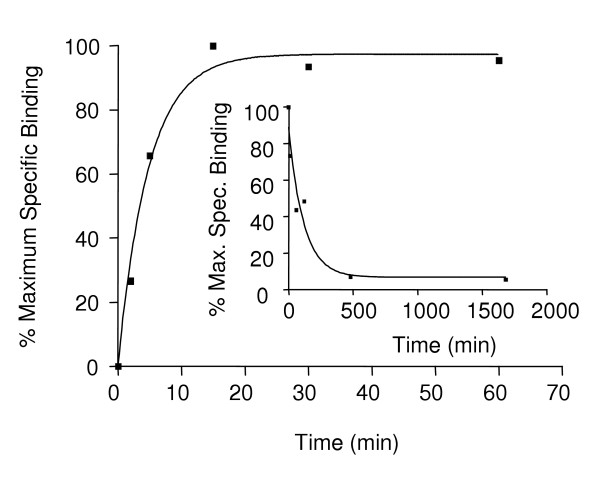
**Association and dissociation (inset) kinetics**. [^3^H]-DHT binding to AR in three-spined stickleback kidney cytosolic extracts was determined at 4°C. The reactions were terminated at time-points ranging between 1 min and 28 h.

Determinations of the binding kinetics in the kidney cytosolic fraction demonstrated rapid association between the ligand and receptor at 4°C. The binding of [^3^H]-DHT had a t_1/2 _of 3.3 min and reached equilibrium after 20 minutes (Fig. 8). The complete dissociation of [^3^H]-DHT bound to the receptor occurred within 8 hours with a t_1/2 _of 80 min (Fig. 8 insert).

### AR steroid specificity

**Figure 9:**
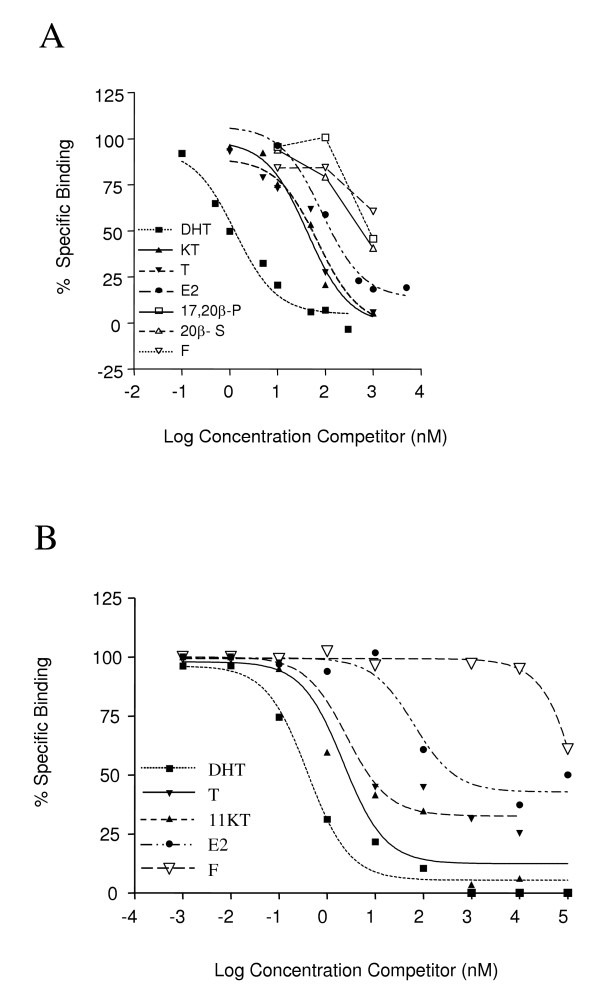
**Competition curves for the binding of various natural steroids to AR in kidney**. *A*, cytosolic extracts, and *B*, reticulocyte lysate samples. Varying concentrations of unlabelled steroids were incubated with 1 nM [^3^H]-DHT over night. Each data point represents the average of two assays. Steroids used were: KT, 11-ketotestosterone; DHT, 5α-dihydrotestosterone; T, testosterone; E2, 17β-estradiol; 17,20β-P, 17,20β-dihydroxy-4-pregnen-3-one; 20β-S, 17,20β,21-trihydroxy-4-pregnen-3-one; F, cortisol.

Ligand competition assays were performed using 1 nM [^3^H]-DHT as a tracer to determine the relative affinity of steroid hormones to AR (Fig. 9). The binding curves were parallel, indicating competitive binding between the various unlabelled steroids and [^3^H]-DHT, allowing EC_50 _values to be determined. Using kidney extracts, the highest affinity binding was observed for DHT (EC_50 _= 1.31 ± 0.18 nM) while both KT and T had lower affinity (42.75 ± 3.42 nM and 68.81 ± 14.45 nM respectively). Binding was also observed for E2 (85.10 ± 17.87 nM), but not for 17,20β-dihydroxy-4-pregnen-3-one (17,20β-P), 17,20β,21-trihydroxy-4-pregnen-3-one (20β-S) or F (Fig 9A). Using ARβ2 containing reticulocyte extract, the highest affinity again was observed for DHT (EC_50 _= 0.67 ± 0.19 nM), followed by KT and T (2.18 ± 0.71 nM and 3.03 ± 0.43 nM), while E2 showed lower binding affinity (64.61 ± 0.28 nM) and F showed poor binding (Fig. 9B). Thus, in both cases DHT was found to have higher affinity than KT or T for the stickleback AR.

### AR regulation

Determination of hormone specific regulation was performed by exposing three-spined sticklebacks for 6 days to DHT, KA, T or E2. None of the three tested androgens regulated AR protein as determined by radioreceptor assay, or AR mRNA as determined by slot blot. However, E2 was found to be a repressor of both AR protein and AR mRNA (data not shown).

### Ligand specific AR gene activation

**Figure 10:**
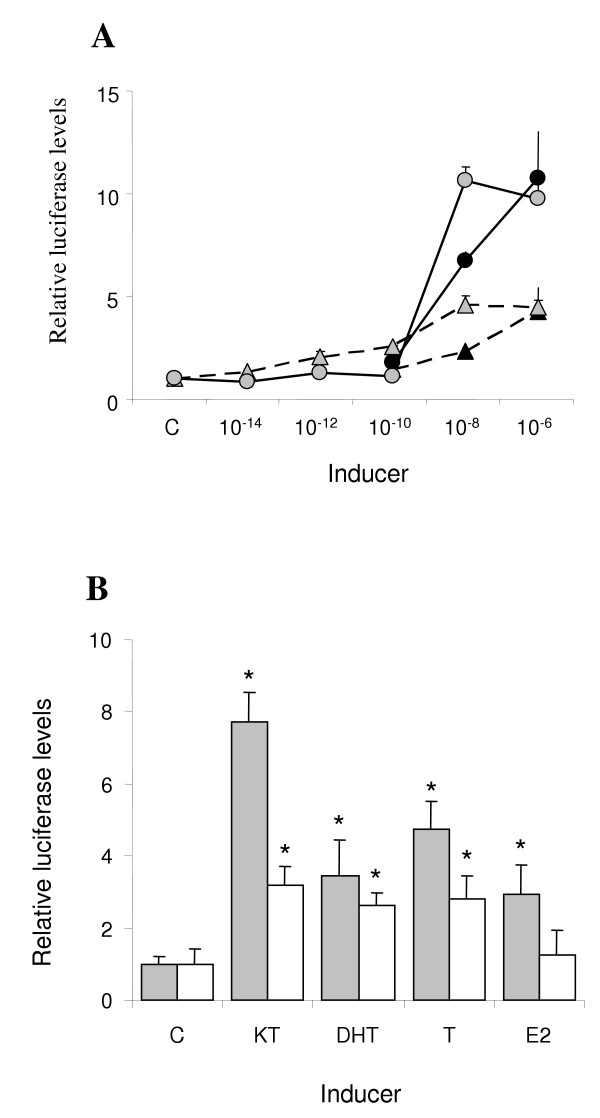
**Activation of stickleback ARβ2 and human AR in transfected cells**. *A*, HepG2 (black) and ZFL (grey) cells were co-transfected with the ARE-luciferase vector, stickleback ARβ2 expression vector and the *Renilla *(pRL) control vector. The cells were treated during 40 hours with increasing concentrations (10^-14 ^M to 10^-6 ^M) of KT (circles) or DHT (triangles). Exposure to the two highest doses (10^-8 ^M and 10^-6 ^M) of both steroids was significantly different (p < 0.01) from the 10^-10 ^M exposure and the control levels. *B*, ZFL cells were cotransfected with the ARE-luciferase vector, the *Renilla *(pRL) control vector, and the stickleback ARβ2 expression vector (grey) or the human AR expression vector (white). The cells were treated with 10^-8 ^M of each steroid. The luciferase levels obtained with KT exposure were significantly different (p < 0.01) from DHT and T when using the stickleback AR but not the human AR expression vector. Statistically significant differences from control levels are indicated with an asterisk (p < 0.01). In both experiments the cells were treated with steroids for 40 h. Data were normalized against the untreated control for each cell line. The results are shown as mean ± SD (n = 4).

The ability of ARβ2 to regulate gene expression through an ARE-regulated luciferase vector was determined using both human HepG2 cells and zebrafish ZFL cells. Both DHT and KT showed dose-dependent activation via the stickleback AR in both HepG2 and ZFL cells (Fig. 10A) The results showed that KT induced luciferase activity 12 fold in ZFL cell and 10 in HepG2 cells, while DHT induced luciferase activity 4.5-fold in ZFL cells and 4.1-fold in HepG2 cells (Fig. 10A). The higher activation obtained with KT was significantly different from the activity obtained with DHT using both 10^-8 ^M (P < 0.001 for both cell lines) and 10^-6 ^M (p < 0.05 for both cell lines) steroid. Furthermore, comparison of the stickleback AR with the human AR, in the ZFL cell line, showed that the stickleback AR was preferentially activated by KT while the human AR did not discriminate between androgens (Fig. 10B).

## Discussion

The aim of the present study was to characterize key components of the AR signaling pathway through which KT mediates its induction of spiggin in the stickleback. Central to androgenic signaling is the interaction between the hormone and its receptor. So far no AR, which is preferentially activated by KT, has been identified in any animal. The only KT-induced gene product clearly identified to date is the underwater adhesive protein, spiggin [11,13], which is synthesized in the kidneys of male three-spined sticklebacks and used to construct a nest. While KT is the main inducer of spiggin production, other androgens will also increase spiggin synthesis in a dose-dependent fashion, although less efficiently. Measurements of androgen levels in stickleback have shown that KT is a prominent androgen in males during the breeding period [14,22]. It has been shown that the KT levels increased from 2 ng/ml during the prespawning period to peak at 40 ng/ml during spawning and to thereafter return to lower levels (≤1 ng/ml) in post spawning male sticklebacks [14,22]. Through these stages the T levels remain at about 1–4 ng/ml plasma. In spawning female stickleback the KT levels have been determined to be 1 ng/ml while the T levels are 20 ng/ml [22]. Circulating DHT levels have not been measured in the stickleback, but are likely to be low since neither DHT nor 5α-androstane-3,17-dione were formed in significant amounts (≤0.5%), if at all, when nesting stickleback testes tissue were incubated with tritiated androstenedione (reexamination of data on which [23] are based). While there are also no measurements of DHT levels in the stickleback kidney, 5α-reductase has been identified in stickleback kidney samples [24], thus suggesting that DHT may be locally formed and functional in stickleback. However, these studies suggest that KT is the main androgen in spawning male sticklebacks. In the present study KA was 10 times more potent than DHT and 50 times more potent than T at inducing spiggin mRNA (Fig. 1). In a previous study, using longer exposures (16 days) spiggin mRNA induction was only observed with KA [13]. These results clearly show that 11-ketoandrogens are by far the most effective inducers of spiggin mRNA. Furthermore, androgen induction of spiggin protein [25] and mRNA synthesis (Olsson, P.-E., Berg, A.H., Lindberg, C., unpublished results) can be blocked by the anti-androgen flutamide. This suggests that spiggin is induced, preferentially by KT, via a traditional AR, belonging to the nuclear steroid hormone receptor family.

Cloning and characterization of the stickleback AR demonstrated that there is a single AR gene locus that codes for a single receptor and that the ligand-binding characteristics of both the cloned and the endogenous receptor are indicative of a single class of high affinity AR (Fig. 7) with higher affinity for DHT than for KT or T (Fig 9). The binding kinetics shows that the AR present in the stickleback kidney has ligand-binding characteristics similar to those of previously characterized nuclear AR from other teleosts [1,2,9].

The stickleback AR shows the greatest similarity to AR from seabream and tilapia, fishes that like the stickleback, but unlike the rainbow trout and eel, belong to Series Percomorpha among the Acanthypterygii [26]. The receptor was found to have 2 splicing variants and 2 poly adenylation sites, and therefore could result in 4 possible different transcripts. The ligand-binding domain of the AR was found to be highly similar to other AR and contains the amino acids that are thought to be important for DHT interaction with the human AR [20,21]. The crystallographic structure of human AR indicates that N705, Q711, R752 and T877 have possible hydrogen bonds with DHT [20]. These 4 amino acids are conserved in teleost AR with one exception; in tilapia ARβ has a R752K substitution (GenBank AB045212). However, no data is available on the function of that receptor. Amino acids L704, M745 and F764 that are considered to have close contact with the ligand in human AR [20], are also conserved in teleost AR. An additional amino acid considered to be important for the architecture of AR is R779 [21]. Three mutations (R779A, R779Q and R779S) of the human AR were tested for trans-activation in COS cells and were found to lead to complete inactivation of AR [21]. It is interesting to note that this amino acid is not conserved among teleosts where both R779S and R779T are found. Neither of these alternative amino acids results in silencing of AR in teleosts as observed by transcriptional activation studies [5,6]. These results demonstrate that the AR ligand binding domains of teleosts and mammals are highly similar, although not identical.

Several previous studies have shown the presence of DHT and T receptors in teleosts, but so far no specific KT receptor has been identified [1,2,5-7]. Comparison of trans-activation efficacy between human AR and Japanese eel ARα and ARβ, using 293 cells, showed that KT was equally effective at activating gene transcription via either the human AR or Japanese eel ARβ [27], and that all three AR failed to distinguish between KT, DHT and T. Although several studies have determined either the ligand binding affinity of AR or the trans-activation of AR, there are few studies dealing with both aspects. However, in one study [8], the rainbow trout ARα ligand binding properties were determined in a heterologous system, the COS-1 cell line. The potency of binding was highest (IC_50_: 6 × 10^-10 ^M), for DHT followed by T, while the poorest binding (IC_50_: 8 × 10^-9 ^M) was observed for KT. In contrast to this, determination of the trans-activation potential using the carp epithelioma papillosum cyprini cell line indicated that the efficiency of activation of the rainbow trout ARα was equal for all three natural androgens [8]. Thus, there was an apparent discrepancy between the binding studies, showing that rainbow trout ARα had higher affinities for DHT and T than KT, and the reporter gene studies, showing that this difference was not reflected in activation potential [8]. In the present study we observed that DHT bound with higher affinity than KT to the native stickleback AR (Fig. 9A) and the *in vitro *translated recombinant stickleback ARβ2 (Fig. 9B). However, following transfection of the stickleback ARβ2-CMVTNT expression vector into either human HepG2 or zebrafish ZFL cells, together with either the *slp*-ARU or the *slp*-ARE, KT was in both cases approximately 2 to 3-fold more potent than DHT in activating the luciferase reporter gene (Fig. 10). In contrast to this the human AR was equally activated by DHT, KT and T (Fig. 10), which is in agreement with earlier studies [27]. Taken together the results are consistent with the data obtained on rainbow trout [8] and suggest that KT preferentially activate the stickleback ARβ2. The N-terminal domain has been suggested to be important for both trans-activation and protein-protein interactions and it is conceivable that there are ligand specific interactions between the LBD and the N-terminal trans-activation domain [28,29]. Evidence that ligand-dependent conformational changes may occur with the AR has been obtained with mesterolone, a synthetic androgen with an additional methyl group on carbon 1 of the A ring. Although mesterolone was found to have a similar binding affinity as DHT and T for both the wild type human AR and a mutant AR found in subfertile men, only mesterolone was able to restore mutant AR function to normal [30].

The 3-fold difference in trans-activation potential probably does not entirely explain the observed potency differences between KT and DHT in inducing spiggin in stickleback *in vivo *[13]. This result may partly be due to the different in the binding affinities of various androgens to the sex steroid binding proteins in fish blood. While KT has low affinity for binding to sex steroid binding proteins [31-36], both DHT and T show high affinities for these binding proteins in both mammals and teleosts [33,35]. The lower binding affinity of 11-ketoandrogens for sex steroid binding proteins in the plasma, and the resulting higher circulating levels of free steroid, may also contribute to the higher effectiveness of KT in inducing spiggin and kidney epithelium hypertrophy as well as male reproductive behavior in three-spined stickleback [12,13,37].

In contrast to estrogen receptors that are auto-regulated by estrogens, AR is not generally up-regulated by androgens and no ARE have been found in the promoter or 5'-flanking region of cloned AR. While AR is generally transcriptionally down regulated by androgens, the protein half-life appears to be increased by androgens [38,39]. Auto-regulation has, however, been observed in some tissues, including rat ventral prostate [40], the Harderian gland located in the orbital cavity of the golden hamster [41], male rat forebrain [42], human bone cells [43] and Atlantic croaker brain [44]. ARE have been identified in the coding region of AR cDNA from rat and shown to be functional, but require interaction with Myc family protein [45]. The ARE sequences (5'-TGTCCT-3') and (5'AGTACTCC-3') are separated by 182 bp in the rat AR cDNA and highly similar sequences are also found in other species, including the three-spined stickleback. However, despite the presence of possible AREs in the AR cDNA we did not observe any auto-regulation of kidney AR by androgens. E2 was the only steroid tested that altered AR mRNA and protein levels. Down-regulation of AR by estrogens is a common feature of AR, and E2 was shown to reduce both the AR mRNA and protein levels of stickleback kidney. This could be a component in the mechanism by which estrogens can suppress kidney hypertrophy in the stickleback [46]. In the present study we found no evidence that upregulation of AR mRNA or protein stability contributes to the KT specificity of spiggin induction. Thus, the stickleback AR is the first example of an AR preferentially activated by KT in any animal. Our results indicate that auto-regulation is not involved in this phenomenon. However, the lower binding affinity of KT than either DHT or T to sex steroid binding proteins in the circulation may be a contributing factor *in vivo*. As these proteins are not present in the *in vitro *systems the presently observed differences in ligand dependent transcriptional activation cannot be due to transport proteins.

### Summary

The present study indicates the presence of a single gene coding for a nuclear AR in the three-spined stickleback. Furthermore, while the results show that the receptor has the highest binding affinity for DHT, it is preferentially activated by KT. While the present study represents the first identification of an AR preferentially regulated by KT, the elucidation of the KT signaling mechanism in teleosts clearly requires further research.

## References

[B1] Sperry TS, Thomas P (1999). Identification of two nuclear androgen receptors in kelp bass (*Paralabrax clathratus*) and their binding affinities for xenobiotics: comparison with Atlantic croaker (*Micropogonias undulatus*) androgen receptors. Biol Reprod.

[B2] Sperry TS, Thomas P (1999). Characterization of two nuclear androgen receptors in Atlantic croaker: comparison of their biochemical properties and binding specificities. Endocrinology.

[B3] Borg B (1994). Androgens in teleost fishes. Comp Biochem Physiol.

[B4] Todo T, Ikeuchi T, Kobayashi T, Nagahama Y (1999). Fish androgen receptor: cDNA cloning, steroid activation of transcription in transfected mammalian cells, and tissue mRNA levels. Biochem Biophys Res Commun.

[B5] Touhata K, Kinoshita M, Tokuda Y, Toyohara H, Sakaguchi M, Yokoyama Y, Yamashita S (1999). Sequence and expression of a cDNA encoding the red seabream androgen receptor. Biochim Biophys Acta.

[B6] Ikeuchi T, Todo T, Kobayashi T, Nagahama Y (1999). cDNA cloning of a novel androgen receptor subtype. J Biol Chem.

[B7] Takeo J, Yamashita S (1999). Two distinct isoforms of cDNA encoding rainbow trout androgen receptors. J Biol Chem.

[B8] Takeo J, Yamashita S (2000). Rainbow trout androgen receptor-alpha fails to distinguish between any of the natural androgens tested in transactivation assay, not just 11-ketotestosterone and testosterone. Gen Comp Endocrinol.

[B9] Sperry TS, Thomas P (2000). Androgen binding profiles of two distinct nuclear androgen receptors in Atlantic croaker (*Micropogonias undulatus*). J Steroid Biochem Mol Biol.

[B10] Wells KL, Van der Kraak G (2000). Differential binding of endogenous steroids and chemicals to androgen receptors in rainbow trout and goldfish. Environ Toxicol Chem.

[B11] Jakobsson S, Borg B, Haux C, Hyllner SJ (1999). An 11-ketotestosterone induced kidney-secreted protein: the nest building glue from male three-spined stickleback, *Gasterosteus aculeatus*. Fish Physiol Biochem.

[B12] Borg B, Antonopoulou E, Andersson E, Carlberg T, Mayer I (1993). Effectiveness of several androgens in stimulating kidney hypertrophy, a secondary sexual character, in castrated male three-spined sticklebacks, *Gasterosteus aculeatus*. Can J Zool.

[B13] Jones I, Lindberg C, Jakobsson S, Hellqvist A, Hellman U, Borg B, Olsson P-E (2001). Molecular cloning and characterization of spiggin. An androgen-regulated extraorganismal adhesive with structural similarities to von Willebrand factor-related proteins. J Biol Chem.

[B14] Mayer I, Borg BV, Schulz R (1990). Seasonal changes in and effects of castration-androgen replacement on the plasma levels of five androgens in the male three-spined stickleback *Gasterosteus acculeatus *L. Gen Comp Endocrinol.

[B15] Jakobsson S, Mayer I, Schulz RW, Blankenstein MA, Borg B (1996). Specific binding of 11-ketotestosterone in an androgen target organ, the kidney of the male three-spined stickleback, *Gasterosteus aculeatus*. Fish Physiol Biochem.

[B16] Sambrook J, Fritsch EF, Maniatis T (1989). Molecular Cloning: A Laboratory Manual.

[B17] Page RDM (1996). An application to display phylogenetic trees on personal computers. Comput Appl Biosci.

[B18] Thompson JD, Higgins DG, Gibson TJ (1994). CLUSTAL W: improving the sensitivity of progressive multiple sequence alignment through sequence weighting, position-specific gap penalties and weight matrix choice. Nucleic Acids Res.

[B19] Verrijdt G, Schauwaers K, Haelens A, Rombauts W, Claessens F (2002). Functional interplay between two response elements with distinct binding characteristics dictates androgen specificity of the mouse sex-limited protein enhancer. J Biol Chem.

[B20] Sack JS, Kish KF, Wang C, Attar RM, Kiefer SE, An Y, Wu GY, Scheffler JE, Salvati ME, Krystek SR, Weinmann R, Einspahr HM (2001). Chrystallographic structures of the ligand-binding domains of the androgen receptor and its T877A mutant complexed with the natural agonist dihydrotestosterone. Proc Natl Acad Sci USA.

[B21] Poujol N, Wurtz J-M, Tahiri B, Lumbroso S, Nicolas J-C, Moras D, Sultan C (2000). Specific recognition of androgens by their nuclear receptor. A structure-function study. J Biol Chem.

[B22] Borg B, Mayer I (1995). Androgens and behaviour in the three-spined stickleback. Behaviour.

[B23] Borg B, Schoonen WGEJ, Lambert JGD (1989). Steroid metabolism in the testes of the breeding and nonbreeding three-spined stickleback, *Gasterosteus aculeatus*. Gen Comp Endocrinol.

[B24] Borg B, Mayer I, Lambert JGD, Granneman J, Schulz R (1992). Metabolism of androstenedione and 11-ketotestosterone in the kidney of the three-spined stickleback, *Gasterosteus aculeatus*. Gen Comp Endocrinol.

[B25] Katsiadaki I, Scott AP, Mayer I (2002). he potential of the three-spined stickleback (*Gasterosteus aculeatus *L.) as a combined biomarker for oestrogens and androgens in European waters. Mar Environ Res.

[B26] Nelson JS (1994). Fishes of the World.

[B27] Ikeuchi T, Todo T, Kobayashi T, Nagahama Y (2001). Two subtypes of androgen and progestogen receptors in fish testes. Comp Biochem Physiol.

[B28] Lee H-J, Chang C (2003). Recent advances in androgen receptor action. Cell Mol Life Sci.

[B29] Reid J, Betney R, Watt K, McEwan IJ (2003). The androgen receptor transactivation domain: the interplay between protein conformation and protein-protein interactions. Biochem Soc Transact.

[B30] Lim J, Ghadessy FJ, Abdullah AA, Pinsky L, Trifiro M, Yong EL (2000). Human androgen receptor mutation disrupts ternary interactions between ligand, receptor domains, and the coactivator TIF2 (transcription intermediary factor 2). Mol Endocrinol.

[B31] Fostier A, Breton B (1975). Binding of steroids by plasma of a teleost: the rainbow trout, *Salmo gairdnerii*. J Steroid Biochem.

[B32] Chang C-F, Lee Y-H, Yoshida T, Sun L-T (1994). Characterization of the plasma sex steroid-binding protein in eel (*Anguilla japonica*). Comp Biochem Physiol.

[B33] Pasmanik M, Callard G (1986). Characteristics of a testosterone-estradiol binding globulin (TEBG) in goldfish serum. Biol Reprod.

[B34] Chang C-F, Lee Y-H (1992). Purification of the sex steroid-binding protein from common carp (*Cyprinus carpio*) plasma. Comp Biochem Physiol.

[B35] Laidley CW, Thomas P (1994). Partial characterization of a sex-steroid binding protein in the spotted seatrout (*Cynoscion nebulosus*). Biol Reprod.

[B36] Øvrevik J, Stenersen J, Nilssen K, Tollefsen K-E (1994). Partial characterization of a sex steroid-binding protein in plasma from arctic charr (*Salvelinus alpinus *L.). Gen Comp Endocrinol.

[B37] Borg B (1987). Stimulation of reproductive behaviour by aromatizable and non-aromatizable androgens in the male three-spined stickleback, *Gasterosteus aculeatus*. Proc V Congr Europ Ichthyol, Stockholm 1985.

[B38] Kemppainen JA, Lane MV, Sar M, Wilson EM (1992). Androgen receptor phosphorylation, turnover, nuclear transport, and transcriptional activation. Specificity for steroids and antihormones. J Biol Chem.

[B39] Zhou ZX, Lane MV, Kemppainen JA, French FS, Wilson EM (1995). Specificity of ligand-dependent androgen receptor stabilization: receptor domain interactions influence ligand dissociation and receptor stability. Mol Endocrinol.

[B40] Mora GR, Mahesh VB (1996). Autoregulation of androgen receptor in rat ventral prostate: involvement of c-fos as a negative regulator. Regulation of androgen receptor mRNA expression in primary culture of Harderian gland cells: cross-talk between steroid hormones. Mol Cell Endocrinol.

[B41] Esposito T, Astore E, Cardone A, Angelini F, Varriale B (2002). Regulation of androgen receptor mRNA expression in primary culture of Harderian gland cells: cross-talk between steroid hormones. Comp Biochem Physiol.

[B42] McAbee MD, DonCarlos LL (1999). Estrogen, but not androgens, regulates androgen receptor messenger ribonucleic acid expression in the developing male rat forebrain. Endocrinology.

[B43] Wiren KM, Zhang X, Chang C, Keenan E, Orwoll ES (1997). Transcriptional up-regulation of the human androgen receptor by androgen in bone cells. Endocrinology.

[B44] Larsson DGJ, Sperry TS, Thomas P (2002). Regulation of androgen receptors in Atlantic croaker brains by testosterone and estradiol. Gen Comp Endocrinol.

[B45] Grad JM, Dai JL, Wu S, Burnstein KL (1999). Multiple androgen response elements and a Myc consensus site in the androgen receptor (AR) coding region are involved in androgen-mediated up-regulation of AR messenger RNA. Mol Endocrinol.

[B46] Oguro C (1957). Notes on the change in the kidney of *Gasterosteus aculeatus aculeatus *(L.) caused by estrogen administration. J Fac Sci Hokkaido Univ Ser VI Zool.

